# The Episodicity of Verbal Reports of Personally Significant Autobiographical Memories: Vividness Correlates with Narrative Text Quality More than with Detailedness or Memory Specificity

**DOI:** 10.3389/fnbeh.2013.00110

**Published:** 2013-08-19

**Authors:** Tilmann Habermas, Verena Diel

**Affiliations:** ^1^Section Psychoanalysis, Department of Psychology, Goethe University Frankfurt, Frankfurt am Main, Germany

**Keywords:** autobiographical memory, episodic memory, narrative, vividness, memory specificity, redemption sequence, autobiographical reasoning, life story

## Abstract

How can we tell from a memory report whether a memory is episodic or not? Vividness is required by many definitions, whereas detailedness, memory specificity, and narrative text type are competing definitions of episodicity used in research. We explored their correlations with vividness in personally significant autobiographical memories to provide evidence to support their relative claim to define episodic memories. In addition, we explored differences between different memory types and text types as well as between memories with different valences. We asked a lifespan sample (*N* = 168) of 8-, 12-, 16-, 20-, 40-, and 65-year-olds of both genders (*N* = 27, 29, 27, 27, 28, 30) to provide brief oral life narratives. These were segmented into thematic memory units. Detailedness of person, place, and time did not correlate with each other or either vividness, memory specificity, or narrative text type. Narrative text type, in contrast, correlated both with vividness and memory specificity, suggesting narrative text type as a good criterion of episodicity. Emotionality turned out to be an even better predictor of vividness. Also, differences between narrative, chronicle, and argument text types and between specific versus more extended and atemporal memories were explored as well as differences between positive, negative, ambivalent, neutral, contamination, and redemption memory reports. It is concluded that temporal sequentiality is a central characteristic of episodic autobiographical memories. Furthermore, it is suggested that the textual quality of memory reports should be taken more seriously, and that evaluation and interpretation are inherent aspects of personally significant memories.

## Introduction

We first sketch the conceptual commonalities and divergences between these three concepts: episodic memory, autobiographical memory, and narrative. From this we derive the principal aim of the study, i.e., to explore the feasibility of some defining properties of the concept of episodic memories by way of testing their intercorrelation, using vividness as an undisputed characteristic of episodicity. Then two additional aims of the study are introduced, namely to explore narrative and other text types and of specific compared to other memory types, as well as to explore differences between memory reports of different evaluative qualities.

Tulving (1972) initially introduced the term episodic memory to separate the domain of traditional memory research on learning of meaningless verbal material, from the emerging field of semantic models of knowledge representation. Episodic memories thus referred to the pairing of contiguous stimuli learned in a one-time experience in the laboratory. Later when Tulving (1983, 1985) moved on to study neurologically impaired patients, he radically shifted the reference of the term episodic memory to include not just the knowledge of something one has learned, but also the remembering of the when and where of learning, and to events of one’s life. Tulving stressed the temporal nature of remembering both in terms of a sense of personal past as well as of the temporal structure of what is being remembered, i.e., the unfolding of an event. At the same time he tied the remembering of past experiences to a specific phenomenal quality, autonoetic consciousness. He defined it as an immediate awareness that the remembered episode has been personally experienced. Other authors use the term “recollective experience” for remembering personal experiences (e.g., Conway, 2009). Because Tulving observed in a neurological patient K. C. that his total retrograde and anterograde amnesia was accompanied by an inability to imagine future actions and scenarios, he expanded the term autonoetic consciousness to include imagining the personal future. Thus the term defines the experience of having an extended existence in time, or a sense of personal continuity (for a narrative critique of this claim see Habermas and Köber, in press). Wheeler et al. (1997) further specified that the episodic memory system enabled individuals to relive past experiences and imagine living through future events. They specified that episodic memory produced the subjective experience of an event, thereby tying episodic memory to the ability to differentiate one’s own subjective perspective from those of others. Remembering from a subjective perspective entails a subjective visual perspective as well as memory of first person experiences such as perceptions, emotions, thoughts, and intentions. Thus at this point the term episodic memory came to include not only remembering, but also imagining hypothetical or future event sequences, deemphasizing the criterion of veridicality.

How can the episodic quality of a memory be judged objectively from a verbal memory report? In an influential paper, Levine et al. (2002) measured the episodic versus semantic quality of verbal reports of autobiographical memories by counting the number of clauses providing information about events, time, place, perceptions, and thoughts and emotions as well as global ratings of the maximum detailedness of the reports. A weakness of these measures is that they strongly depend on the length of the memory report, although the global ratings of detailedness, which were also used, do so to a far lesser degree.

A second tradition of memory research evolved in parallel to the concept of episodic memory. Neisser (1982) pleaded for expanding the scope of memory research to memories of personal experiences from one’s life. The term autobiographical memory (Robinson, 1986) was used for personal memories that are related to the development of the self. This could be seen in the reminiscence bump (Rubin et al., 1986), i.e., an oversampling of memories from adolescence and young adulthood in adults over age 40, which was related to adolescent identity development (Fitzgerald, 1988). Neisser (1986) suggested that autobiographical memory has a hierarchical, nested structure. Barsalou (1988) found that besides specific events taking place at a specific date autobiographical memory reports may also contain recurrent (“summarized”) events and extended events that cover several days or weeks, such as a trip, both termed “generalized events.” In Conway’s (1990) hierarchical model of the structure of autobiographical memory event specific knowledge is nested in generalized events, which are nested in extended time lines covering longer periods of life, which in turn are integrated by the life story schema (Bluck and Habermas, 2000; Conway et al., 2004). The autobiographical memory system is closely tied to mental representations of the self (Conway and Pleydell-Pearce, 2000).

Thus autobiographical memory covers both autobiographical knowledge as well as autobiographical memories of varying temporal extension. In this terminology, specific events or specific memories overlap with Tulving’s later understanding of episodic memories, although the memory of a specific event such as a birthday party may either just be known or actually be remembered as a sequence of perceived events, i.e., an episodic memory. Parts of this tradition stress the temporal specificity and limited extension of an event more than a sense of reliving or of personal continuity (for exceptions see, e.g., Conway, 2009). The measure of episodic versus semantic quality of autobiographical memories suggested by Kopelman et al. (1989) is closer to this tradition, as the episodicity of a memory is rated on a four-point scale ranging from specific events with temporal and locational details to no response or an answer based on knowledge.

A third, linguistic tradition may also contribute to an understanding of remembering past experiences, since these are communicated in language. This tradition categorizes types of texts according to their function and form. Narrative is a text type which serves to communicate an event by imitating the sequence of events, typically in sentences beginning “and then …, and then …, and then.” Labov and Waletzky (1967) suggest that narrative clauses are at least two sequential main clauses, the referential meaning of which would change if their order was reversed. For instance, the clauses “John was hungry. John had dinner at the local restaurant” change meaning if their order was reversed. Instead of John going to the restaurant to appease his hunger, the sentences would then imply that the restaurant served portions too small to satisfy John’s hunger. Labov and Waletzky point out that narrative texts have a second main function besides imitating an event sequence, namely that of evaluating the events. Evaluations are always made from a specific perspective, either by one of the participants of the event or from without, e.g., by the narrator. All mental expressions referring to a subjective point of view including all comments and interpretations by the narrator or any other individual are termed evaluations. Accordingly, narrative texts comprise not only narrative clauses, but also other kinds of clauses. When the narrator evaluates a past event by expressing an emotion, explaining or contextualizing the event, this is done in non-narrative clauses. Other text types that may be used in the context of remembering are chronicles (Linde, 1993) that summarize temporally structured events without imitating the sequence of events, and arguments that explain or justify statements (Rosenthal, 1995). If a text contains at least two narrative clauses, the text is considered a narrative (Labov and Waletzky, 1967), even though it typically also contains clauses with chronicles, arguments, and descriptions.

Episodic memories are most adequately communicated in narrative form, because specific episodes are temporally structured events. Reliving an event implies following the sequence of events. Actually narrating an event renders it easier to relive an episode than merely imagining it, because the narrative format requires a temporal and causal ordering of events. Narrative clauses, use of the protagonist’s subjective perceptual perspective, and means of dramatic narrating such as historic present and direct speech facilitate a reliving for both narrator and listener (Nelson et al., 2008; Habermas and Diel, 2010). The use of emotion words is not necessary for a vivid narrative, as emotions are most directly elicited by the event sequence itself.

Comparing the narrative format to episodic memories, narratives need not be limited to a short duration or precise location, nor need they be told from the narrator’s past perspective, but may also be narrated from someone else’s or an objective, outside perspective. Also, although specific memories tend to be narrated and narratives tend to concern specific memories, these need not be narrated, but may be summarized. On the other hand, also extended events can be narrated, although usually there is a highly specific event at the core of a narrative, a complicating event.

In this paper we argue that the narrative format appears to best describe the textual form of reports of the subjective experience of remembering and reliving an event. We suggest that narrative is the textual form of reports of all subjective experiences covered by Tulving’s term autonoetic experience, which characterizes episodic memory. Similar to Tulving’s definition, we deemphasize the role of details, which are important to measure the degree of veridicality of a memory. Instead we focus on the episodic, i.e., story-like nature of episodic memories which differentiates them from mere knowledge. Therefore a narrative approach agrees with Tulving that some non-autobiographical memories and experiences are to be included in the definition of episodic memories, such as memories of fictional stories. In this sense, as in everyday language use (episodic) memories are such irrespective of whether they regard personal experiences (autobiographical) or fictional stories, whether they are true or not.

Possible arguments for conceptually grouping episodic autobiographical memories with episodic memories of fictional or reported stories and with the experiences of daydreaming and anticipating future scenarios may be that all of these phenomena are simultaneously affected by a specific neurological damage, or that they activate the same brain regions, or similarities in the quality of subjective experience (Tulving, 2002). However, we argue that the subjective experience of reliving corresponds to a specific format, which is needed to communicate this experience, i.e., narrative. Under ideal conditions that minimize the probability of distortions, a narrative communication reflects a subjective experience of remembering or imaginatively living through an event. The narrative format of a verbal report obtained under ideal conditions is the best way to objectively judge the episodicity of memories and experiences. If this was true, the narrative quality of memory reports should correlate with another symptom of reliving, namely the vividness of a report.

In our study we set out to provide this evidence, comparing the empirical relation between vividness and aspects of the three definitions of remembering of personal experiences. We study these in the most personally significant memories possible, i.e., the ones included in life narratives. Therefore in adults these autobiographical memories are also most likely to be contextualized in the narrator’s life to elaborate their biographical significance. This process of “autobiographical reasoning” (Habermas and Bluck, 2000; Habermas, 2011) is more than mere remembering of past experiences, and has been discarded as “external detail” in research on episodic memory (Levine et al., 2002). It is the essential way of processing autobiographical memories by tying them to other parts of life and to the development of the self. Thus life narratives contain both autobiographical memories and their evaluative, interpretative elaboration.

Whereas in earlier analyses (Habermas and de Silveira, 2008) we were interested in the global coherence of life narratives, here we segmented life narratives into thematic units comparable to memories to render comparison to research on isolated episodic memories easier. We then coded each segment separately for text type and memory specificity, and we rated detailedness of indications of time, place, and persons as approximations of the three definitions of remembering events from the personal past. We also rated vividness as an aspect that can be related to all three approaches.

The main aim of this study was to test how some of the suggested aspects of episodic autobiographical memories are related to vividness as a relatively undisputed characteristic of episodic memories. We assume that suggested aspects of episodic memories need to be correlated to vividness and other aspects that define episodic memories if they are to be accepted as defining or typical aspects. Thus if suggested criteria of the episodicity of memories fail to correlate with vividness and with other criteria, we suggest discarding them as defining elements of the episodicity of memories. Our expectation was that out of the three characteristics of personal memories, narrative is the best predictor of vividness because it establishes a gradual unfolding of the past sequence of events. This expectation is supported by the observation that when asked to tell familiar fairy tales, the aforementioned amnesic patient K. C. was able to name a relatively normal number of themes present in the tales, but failed to establish the correct order of these themes, offering disorganized, discoherent accounts (Rosenbaum et al., 2009). This does not point to a lack of either (semantic) knowledge or of memories of specific events as represented by the themes, but specifically to a lack of the ability to provide narrative structure to events. Finally, we expected the evaluation of past events in terms of emotions, interpretation, and integration of the event into the life story to be less related to vividness.

A second aim of the study was to explore differences between narrative and other text types and between specific and other kinds of memories. Given the current focus on episodic autobiographical memories, there is little research on other than specific types of memories and other than narrative types of text. Memories of recurrent events, for example, are relatively frequent in autobiographical accounts. In contrast to specific memories which tend to focus on the exceptional (Bruner, 1990), memories of repeated events may serve to underline the typical and habitual. For instance, when talking about one’s childhood, sentences beginning with “We used to …” provide a sense of what it was like back then. A recent comparison of memory types evidenced that memories of recurrent events served more social functions, whereas specific and extended memories serve more self and directive functions (Waters et al., 2013). We expected both specific memories and narrative texts to be the most vivid and most detailed categories, chronicles and generalized event memories next, and arguments and memories of extended periods and comments last.

A third aim of the study was to explore differences between memory reports of differing valence. Negative events tend to be narrated more fully (Habermas et al., 2009b). Also, avoidance of specific memories and preference of repeated or extended events is typical for depressed individuals who may wish not to confront the specifics of negative events (Williams et al., 2007), which again suggests that specific memories are more negative than generalized memories or memories of entire periods.

We were interested not only in comparing positive with negative memories, but also in memories of mixed valence and especially in memories in which there is a change of valence. McAdams (2006) termed episodes in which valence shifts from bad to good redemption sequences, and episodes in which good turns bad contamination sequences. Redemption sequences are related to resilience, contamination sequences to depression (Lilgendahl and McAdams, 2011). The inclusion of sequences of changing valence pays tribute to the temporally sequential nature of (memory) narratives, which canonically start with a (positive or neutral) normal state of affairs, then present a problem or complication, followed by attempts to solve it and a positive or negative outcome. Thus memories, especially if they are remembered sequentially like in a narrative, supporting reliving, are not always homogenous in valence. Rather complications usually provoke negative evaluations and emotions, while the final result may be a happy or not so happy end (cf. Habermas and Berger, 2011). We were interested in exploring differences between events of homogeneous and of mixed valence. This interest results from taking seriously the temporally sequential nature of episodic autobiographical memories, which is part of Tulving’s later definition of episodic memory, but often not considered empirically.

Our data set had originally been collected to answer developmental questions. The four younger age groups have been used to study the development of global coherence in life narratives (Habermas and de Silveira, 2008; Habermas et al., 2009a). Some of the ratings and codings presented here have been used in a developmental analysis of the lifespan development of episodic memory and autobiographical reasoning (Habermas et al., in press). In this paper we do not pursue developmental interests. Rather, the extant age range of the participants serves to ensure that results can be generalized across almost the entire life span. The only age-related limitation is that autobiographical reasoning, reflected in the code life story integration, increases across adolescence (Habermas and de Silveira, 2008), and that the amount of detail and length of memory reports increase slightly between ages 8 and 16 (Willoughby et al., 2012) also in this sample.

## Materials and Methods

### Participants

A total of 168 females and males from a central German city was distributed across six age groups (8, 12, 16, 20, 40, 65 years) with mean ages of 8.57 years (SD = 0.28, 14 girls, 13 boys), 12.38 (0.36, 14, and 15), 16.61 (0.43, 14, and 13), 20.52 (0.53, 13, and 14), 40.79 (2.87, 14, and 14), and 64.53 (2.27, 15, and 15). The youngest age group was the higher achieving half of third graders from a grade school, the adolescent and young adult groups were students of a Gymnasium, leading up to an “Abitur” which allows entry into university. Participants for the two oldest age groups were recruited with flyers distributed widely in local shops, at sports facilities, doctors’ offices, and among continuing education University students. The younger age groups were heading toward an “Abitur,” whereas 24 of the middle-aged and 19 of the older adults had “Abitur” or “Fachabitur,” and 4 and 9 respectively had finished school after 10 years, with 2 of the older adults with a lower or no school degree. In the middle-aged group 14 held a College degree, 17 in the oldest group. Thus the four younger age groups were well-educated, and the educational level of the two older age groups was also well above average. Informed consent was obtained from all participants and from the parents of participants younger than 18 years.

### Procedure and materials

The three younger age groups were interviewed individually by one of five different trained female interviewers in their mid-twenties in their schools, the three older age groups in our lab. Participants wrote seven most important memories from their lives each on a card, dated them, and put them in sequence on the table. Then they narrated their life in 15 min, integrating the seven memories and explaining how they have become the person they are today (cf. Habermas and de Silveira, 2008, for verbatim instructions). Participants were not interrupted except for a reminder of the time left after 10 min, and otherwise only encouraged to continue non-verbally. The seven memories served to ensure that specific episodes were integrated into the life story.

#### Segmenting

Verbatim transcripts were created from audio-files. They were divided into thematic segments containing at least four clauses (Diel et al., 2007). The prototype of a segment is a narrative focusing on a specific, datable event. Ideally segments are explicitly introduced and ended. Two research assistants independently segmented 16 entire life narratives κ = 0.82. Then each coder segmented half of the remaining narratives. To check the quality of the ensuing segmenting, another randomly chosen 16 narratives were coded also by the respective other coder, yielding *K* = 0.92. A deviation of a segment border of up to one clause was tolerated.

#### Coding

Segments served as basic units for coding, each segment receiving a code or rating (Diel et al., 2009). Ratings were made on scales ranging from 0 to 3 following Levine et al. (2002). Each point was defined in a manual. In the Appendix we present three examples of segments with their codes and ratings to illustrative purposes. For each participant, mean ratings and relative frequencies of codes were used. To measure interrater reliability, we used single intraclass correlations for ratings and Cohen’s Kappa for nominal codes.

##### Detailedness of person, place, and time

Three aspects of the detailedness of information regarding persons, place, and time were rated. Person detail received a 3 if an individual was depicted very graphically with at least three details, a 2 if an individual was described with at least one additional information, a 1 if an individual was named, and a 0 if no or only anonymous people were mentioned. Place detail received a 3 if the location was precisely determined, e.g., by naming a specific building, a 2 if a location was named without allowing the identification of a precise place (e.g., the name of a long street), a 1 if only a generic place or a town was named, and a 0 if no location was named. Temporal detail was rated with a 3 if a date was provided, a 2 if a at least a year was provided, a 1 if the temporal indications were relatively imprecise, and a 0 if no temporal information was included that would allow a listener to roughly know when the event had taken place. Two raters independently rated person detail in 16 entire life narratives (261 segments), *r*_ic_ = 0.83. Then one of the two rated the remaining narratives. To check again the quality of the ensuing coding, another eight of these remaining narratives (140 segments) were rated also by the other rater, yielding *r*_ic_ = 0.80. Intraclass correlation for place detail was *r*_ic_ = 0.86 (0.82) and for time detail *r*_ic_ = 0.84 (0.87).

##### Vividness and emotions

For vividness, a value of 3 was given if the overall impression was very vivid and the segment included a dramatized narration of a specific event, a 2 if the segment was fairly vivid and contained intensifiers like “very” and global evaluations like “it was just great,” a 1 if the segment was not vivid and at the most one emotion label, global evaluation, or intensifier, and a 0 if the segment appeared dry and monotonous. Interrater reliabilities were *r*_ic_ = 0.82 (0.81). For emotions, 3 was rated if several emotions were named and at least one emotion was described in detail, a 2 if several emotions were only named, but not further specified, or if one emotion was named and elaborated, a 1 if one emotion was named, and a 0 if no emotion was named. Interrater reliabilities were *r*_ic_ = 0.85 (0.84) based on 261 (140) segments.

##### Text type and memory type

We coded two typologies of memory reports: memory specificity was taken from memory psychology and text type taken from linguistics. Although specificity aims at the content, text type at the form of text, they still should be highly related, because if specific events are talked about at length, they tend to be narrated. Following Barsalou (1988), we coded segments either as containing a specific memory of an event lasting up to a day, as generalized event, i.e., repeated events or events extending between a day and a year, as a period lasting longer than a year [approximating Conway’s (1990) “lifetime period”], or as a segment that does not refer to a temporal unit, such as a comment. Interrater reliabilities were κ = 0.81 (0.71) based on 261 (140) segments.

Additionally, segments were coded as a narrative text if it contained at least two consecutive narrative clauses referring to consecutive events (Labov and Waletzky, 1967), as a chronicle, if it was not coded as narrative and the main bulk of the segment summarized events (Linde, 1993), or as an argument, if it was not coded as a narrative and the main part of the segment contained evaluations or interpretations (Rosenthal, 1995). Interrater reliabilities were κ = 0.83 (0.79) based on 250 (140) segments.

##### Life story integration and interpretation

We rated the degree to which the segment was related to other parts of the life story and the degree to which it was interpreted. Life story integration was rated 3 if the segment contained at least three references to other times or to other topics in life, 2 for two, 1 for one, and 0 for none such reference. Interpretation was rated 3 if the significance of the segment for one’s life, a change in personality, or a profound change of attitude was described, 2 if the narrator described her or his own personality, explained emotional reactions, or gave a meaning to the event, 1 if an event was evaluated positively or negatively or emotions were mentioned, and 0 if events were only factually narrated, but not evaluated. Life story integration achieved an interrater reliability of *r*_ic_ = 0.81 (0.31), interpretation of *r*_ic_ = 0.82 (0.60), based on 337 (140) segments.

##### Valence

We coded the overall valence of the segment with six categories: neutral, ambivalent, positive, negative, initially negative turns out well (redemption sequence), and initially good turns bad (contamination sequence; McAdams, 2006). Interrater reliabilities were κ = 0.80 (0.75) based on 250 (140) segments. We also constructed a continuous variable of valence (used in Table 1), assigning −2 to contamination, −1 to negative valence, 0 to neutral and ambivalent segments, 1 to positive segments, and 2 to redemption sequences.

**Table 1:** **Correlations between aspects of memory reports (*N* = 3075 segments, upper right triangle) and means of correlations within participants (*N* = 168 participants, lower left triangle; significant correlations in bold)**.

		1	2	3	4	5	6	7	8	9	10	11	12
1	Vividness		0.13	−0.04	0.00	0.03	0.28	0.49	0.38	0.18	−0.01	−0.15	0.46
2	Detail of person	0.11		0.07	0.02	−0.02	0.04	0.09	0.02	0.17	−0.04	−0.01	0.20
3	Detail of place	0.01	0.06		0.09	0.00	0.02	−0.06	−0.07	0.08	0.03	0.22	0.13
4	Detail of date	−0.04	0.03	0.08		−0.02	−0.07	0.00	0.01	0.19	0.06	0.01	0.07
5	Specific memory	−0.01	−0.02	−0.01	0.01		0.30	0.07	0.00	−0.05	−0.04	−0.06	0.06
6	Narrative text	**0.28**	0.08	0.02	−0.08	**0.29**		0.13	0.00	0.02	−0.06	−0.10	0.31
7	Emotion	**0.60**	0.07	−0.03	−0.02	0.02	0.02		0.25	0.13	−0.05	−0.09	0.31
8	Interpretation	**0.45**	0.03	−0.04	−0.02	0.04	0.07	**0.32**		0.18	0.02	−0.02	0.32
9	Life story integr.	**0.19**	**0.19**	0.04	0.18	−0.04	0.03	0.15	**0.16**		0.01	0.02	0.35
10	Valence	0.01	−0.04	0.08	0.03	−0.05	−0.05	−0.05	0.01	0.05		−0.06	−0.05
11	Age of memory	−0.13	0.08	0.05	0.05	**0.17**	−0.03	−0.13	−0.08	−0.02	−0.05		−0.02
12	Segment length	**0.52**	**0.23**	**0.23**	0.05	0.08	**0.37**	**0.37**	**0.35**	**0.38**	−0.03	−0.11	

*Only for mean correlations within participants (lower left triangle): p < 0.05 if r > 0.15, p < 0.01 if r > 0.19, p < 0.001 if r > 0.27*.

##### Age of memory

The age of each segment was judged in terms of age at the beginning and age at the end of the period covered by the segment, which were then averaged. Interrater reliabilities were *r*_ic_ = 0.94 (0.95) for the averaged values used here, based on 15 life narratives with 249 segments (5 life narratives, 88 segments).

## Results

Descriptive statistics at the level of individuals use ANOVAs. Since we are interested in relations between different aspects of life narrative segments, we analyze at the level of segments. In addition, because a varying number of several segments belongs to the same life narrative by the same narrator (*n* = 3075, *N* = 168), segments are not independent measurements. Therefore we mostly provide measures of the magnitude of differences or correlations. To provide some information about probabilities, we will aggregate correlations for each individual to test hypothesis 1. We will comment on correlations not under 0.15 and effect sizes indicating a prediction of variance of not below 2%. The corresponding correlation of 0.15 marks the lower limit of half of all correlations considered in a large set of meta-analytic studies (Hemphill, 2003). This lower limit also acknowledges that variables with only two to four points tend to result in relatively low correlations. This approach is tuned to the object of interest, i.e., qualities of segments measured repeatedly in varying numbers for each individual. It is also in line with the more exploratory nature of the study as well as with the recent stress on magnitude of effects rather than their probability.

### Data description

Life narratives consisted of a mean of 241 propositions (*SD* = 103.20) and 18.31 segments (*SD* = 6.90). Segments contained a mean of 13.37 propositions (*SD* = 3.57). In ANOVAs with age group and gender as factors, length of life narratives both in terms of overall number of propositions [*F*(5, 156) = 9.94, *p* = 0.000, η^2^ = 0.24] and number of segments [*F*(5, 156) = 10.13, *p* = 0.000, η^2^ = 0.25] significantly varied with age, as did, to a lesser degree, mean number of propositions per segment [*F*(5, 156) = 2.71, *p* = 0.022, η^2^ = 0.08]. Increase in length of entire narratives and of segments was linear with age, with the exception of the 40-year-olds for life narrative length and an exception of the 65-year-olds for segment length. There were no overall gender differences.

### Correlations between possible indicators of episodicity

To explore the relation between possible indicators of episodicity, we calculated all correlations on the basis of all segments (*N* = 3075; Table 1, upper right triangle). To enable us to test the significance of correlations and of differences between them, we also calculated all correlations within individuals, then transformed them to Fisher’s *z*-values, and then averaged them across individuals (Table 1, lower left triangle). Among the correlations between aspects of memory reports which have been proposed as aspects of the episodicity of memories, i.e., the first six aspects in Table 1, there was an expected correlation between narrative and memory specificity (*r* = 0.29) and between narrative and vividness (*r* = 0.28). All the other correlations between the first six variables are negligible. Thus the measures of detailedness of persons, place, and time correlated neither with each other nor with vividness, narrativity, or memory specificity. To compare correlations with vividness, we calculated *t*-tests for dependent correlations. Narrativity indeed correlated significantly more with vividness than memory specificity, *t*(134) = 2.97, *p* < 0.01 and also more than detailedness, *t*(158) = 1.98, *p* < 0.05.

Segment length correlated most with vividness, and also, to a lesser degree, with narrativity, emotionality, interpretation, and life story integration. Related aspects correlated with each other, such as vividness and emotionality, and interpretation and life story integration. Unexpectedly, interpretation correlated strongly with vividness, and, to a lesser degree, with emotionality. Separate correlations for the three pairs of adjoining age groups revealed the same pattern of correlations. Only correlations with life story integration were larger in older age groups, due to the near absence of life story integration in lower age groups (cf. Habermas and de Silveira, 2008; Habermas et al., in press).

For descriptive purposes, we also ran a principal components analysis with correlations between all segments. Two components resulted from a screen test, explaining 22.2 and 13.1% of variance respectively. After varimax rotation the first component had loadings of 0.79 for vividness, 0.77 length, 0.64 emotion, 0.55 interpretation, 0.46 life story integration, and 0.40 narrative text format. Component 2 had loadings of 0.53 for life story integration, 0.50 for detail of place, 0.48 for temporal detail, −0.44 for narrativity and −0.41 for specificity, and 0.40 for age of memory. All other loadings were below 0.3. The first component may be interpreted to assemble characteristics of the episodicity of the verbal report, with a surprising, though low loading of life story integration. The second component may be interpreted to represent aspects of verbal reports that help locate events in life, in time, in space, which plausibly appears to be more necessary for older memories.

### Concurrent prediction of vividness as proxy for episodicity

Taking vividness as the most uncontroversial proxy for episodicity of a memory report among the variables coded here, we explored the simultaneous correlations of possible indicators of episodicity on vividness. To this end we ran three stepwise regressions on vividness, entering all variables that explain more than 1% of variance to explore the variables’ relative contributions in predicting vividness (*N* = 3075 segments). First we used only the five possible indicators of episodicity. Narrative text format predicted 7.9% of variance (β = 0.28), person detail added another 1.4% (corrected *R*^2^ = 0.091, Δ*R*^2^ = 0.014, β = 0.12). However, when segment length was added, it predicted 21.5% of variance (β = 0.46), reducing the contribution of narrative text to 2% (corrected *R*^2^ = 0.235, Δ*R*^2^ = 0.020, β = 0.15). Entering all variables in a third regression analysis left this contribution of narrative text intact: emotionality (corrected *R*^2^ = 0.242, β = 0.49), segment length (corrected *R*^2^ = 0.350, Δ*R*^2^ = 0.108, β = 0.35), interpretation (corrected *R*^2^ = 0.383, Δ*R*^2^ = 0.034, β = 0.20), and narrative text (corrected *R*^2^ = 0.406, Δ*R*^2^ = 0.023, β = 0.16). Out of the five suggested indicators of episodicity, narrative text format is still the best predictor of vividness even when used concurrently with segment length and all other variables to predict vividness. However, the effect size of narrative text format is reduced substantially by adding segment length from 8% to only 2%.

The unexpected correlation between vividness and interpretation might have to do with the way we operationalized interpretation: a 1 had been assigned if an emotion was not just transported by the plot, but was actually put in words and named. This is a very basic way of interpreting lived experience and does not carry with it the cognitive and distancing flavor stronger interpretation does. Also this definition of a rating of 1 for interpretation is similar to how a 1 for emotion was assigned. This explanation of the correlation between vividness and interpretation was confirmed when comparing mean vividness ratings for segments with interpretation values of 0, 1, and 2 or 3: while an interpretation rating of 0 was low in vividness (*M* = 0.77, SD = 0.63), there was no difference in vividness between an interpretation rating of 1 (*M* = 1.68, SD = 0.73) and 2 or 3 (*M* = 1.66, SD = 0.78). Accordingly separate correlations between vividness and a dichotomous variable with a 1 for a 1 in interpretation and a 0 for all other interpretation ratings was higher (*r* = 0.28) than between vividness and a dichotomous variable with a 0 for interpretation ratings below 2 and a 1 for interpretation ratings of 2 or 3 (*r* = 0.18). The reverse pattern showed for correlations of the two dichotomous interpretation variables with life story integration (*r* = 0.16). Thus the correlation between vividness and interpretation is driven by the naming of emotions, but not by stronger forms of interpretation.

### Differences between text types and memory types

We explored differences between text types and memory types, focusing on narratives and specific memories as the types that come closest to specific episodic autobiographical memories. Out of a total of 3075 segments, 34.8% were narratives, 33.1% chronicles, and the remaining 32.1% arguments. Regarding memory types, 17.4% were specific memories, 49.7% extended events lasting more than a day and up to a year, 19.7% periods lasting over a year, and 13.2% atemporal comments (Table 2). Of the specific memories 66.3% were narrations, and 33.1% of narratives regarded a specific memory. Thus most specific memories tend to be narrated, but narratives may also cover longer time stretches.

**Table 2:** **Crosstabulation of text type with memory types**.

Text type	Memory type – event				
	Specific	Generalized	Long	Comment	Total
Narrative	354	494	124	97	1069
	*16.8*	*−2.8*	*−8.3*	*−4.9*	
Chronicle	94	565	268	91	1018
	*−8.2*	*4.5*	*6.5*	*−4.9*	
Argument	86	470	215	217	988
	*−8.7*	*−1.6*	*1.9*	*9.9*	
Total	534	1529	607	405	3075

*Rows in italics indicate corrected residuals per cell*.

Text type explained more variance of ratings and length than did memory type (Table 3). Most notably, text type explained 8% of variance in length of segment and 6% of variance in vividness. Narrative was the longest and most vivid text type. Memory type, in contrast, only explained 4% of variance of detailedness of persons. Comments were most detailed regarding persons, apparently regarding comments on enduring aspects of individuals. Although on average specific memories were the longest and the most vivid segments, these differences did not account for more than 0.5 and 0.3% of variance respectively. It is interesting to note that both in terms of text type and memory type, detailedness tended to be greatest in segments summarizing events and covering long stretches of time or repeated events. Thus detailedness in terms of person, place, and time was not a characteristic of narrative nor specific memories.

**Table 3:** **Text types, memory types, and valence (means, standard deviations in italics, effect sizes)**.

	Vividness	Detailedness			Life story integration	Interpretation	Emotion	Text length
		Person	Place	Time	
Total	1.44	1.21	0.91	1.11	0.65	1.11	0.56	13.20
	*0.82*	*0.78*	*0.72*	*0.91*	*0.73*	*0.91*	*0.86*	*8.59*
**TEXT TYPE**								
Narrative	1.75	1.25	0.95	1.02	0.62	1.11	0.71	16.9
	*0.87*	*0.71*	*0.67*	*0.91*	*0.74*	*0.87*	*0.95*	*10.5*
Chronicle	1.27	1.19	1.06	1.29	0.77	1.07	0.49	12.0
	*0.75*	*0.79*	*0.71*	*0.87*	*0.77*	*0.93*	*0.81*	*6.8*
Argument	1.26	1.19	0.75	1.02	0.54	1.14	0.47	10.5
	*0.73*	*0.84*	*0.71*	*0.93*	*0.65*	*0.92*	*0.80*	*6.3*
η^2^	0.06	0.01	0.02	0.01	0.01	0.00	0.01	0.08
**MEMORY TYPE**								
Specific	1.49	1.17	0.92	1.07	0.57	1.09	0.69	14.4
	*0.85*	*0.65*	*0.70*	*1.00*	*0.68*	*0.84*	*0.92*	*9.4*
Generalized	1.46	1.14	0.91	1.10	0.61	1.10	0.54	12.9
	*0.80*	*0.76*	*0.68*	*0.90*	*0.72*	*0.92*	*0.85*	*8.4*
Long period	1.38	1.20	1.10	1.31	0.80	1.14	0.49	13.4
	*0.85*	*0.80*	*0.70*	*0.80*	*0.77*	*0.94*	*0.82*	*8.6*
Comment	1.38	1.57	0.72	0.90	0.67	1.08	0.56	12.5
	*0.81*	*0.88*	*0.74*	*0.96*	*0.74*	*0.92*	*0.86*	*8.3*
η^2^	0.00	0.04	0.02	0.01	0.01	0.00	0.00	0.01
**IMPLICIT VALENCE**								
Neutral	0.81	1.15	1.00	1.23	0.50	0.69	0.14	9.7
	*0.74*	*0.81*	*0.78*	*0.97*	*0.65*	*0.91*	*0.47*	*5.5*
Positive	1.41	1.18	0.94	1.14	0.60	1.11	0.44	12.0
	*0.78*	*0.78*	*0.71*	*0.92*	*0.71*	*0.91*	*0.74*	*7.2*
Negative	1.52	1.24	0.90	0.98	0.61	1.12	0.69	13.8
	*0.83*	*0.76*	*0.69*	*0.89*	*0.72*	*0.87*	*0.94*	*9.1*
Ambivalence	1.56	1.32	0.88	1.17	0.75	1.23	0.68	15.6
	*0.80*	*0.78*	*0.69*	*0.90*	*0.77*	*0.92*	−*0.93*	*9.9*
Redemption	1.65	1.17	0.94	1.13	0.85	1.25	0.82	15.5
	*0.83*	*0.74*	*0.70*	*0.90*	*0.75*	*0.95*	*1.01*	*10.0*
Contamination	1.59	1.25	0.84	1.03	0.80	1.09	0.74	15.4
	*0.75*	*0.82*	*0.66*	*0.83*	*0.79*	*0.77*	*0.99*	*10.2*
η^2^	0.07	0.00	0.00	0.01	0.02	0.02	0.05	0.04

### Valence: Positive and negative segments, redemption, and contamination sequences

Finally we explored whether events of differing valence also differed in other respects. All segments were coded for valence, with 40.4% positive segments, 23.0% negative, 13.2% ambivalent, and 8.8% neutral, and with 10.0% redemption sequences in which an initially negative state turned out well, and 4.6% contamination sequences in which an initially positive state turned out negative.

Segment valence explains the most variance in ratings and length (Table 3) regarding vividness (7%), emotion (5%), and length of segment (4%), and to a lesser degree, also some variance of interpretation and life story integration (2%). The general trend with all these aspects is that redemption, then contamination and ambivalent segments are longest, most vivid and emotional, and also most interpreted and linked to other parts of life, followed by negative, then positive, and finally neutral segments. Thus segments in which there is a sequence of actions with a change of fortune are, not surprisingly, the most vivid and emotional texts, followed by texts with more stationary mixed emotions.

With regard to the contingencies of valence with text type and memory type (Table 4), negative segments were most frequently narrated as expected, redemption sequences were more often narratives or chronicles, and contamination sequences were most often chronicles, followed by narratives. A similar, but less pronounced pattern showed in memory types. Negative segments were most frequently found in specific memories, redemption sequences in specific and extended memories, and contamination sequences in extended events.

**Table 4:** **Crosstabulation. of evaluative patterns with text and memory types**.

Evaluative pattern	Memory type – event			
	Specific	Generalized	Long	Comment
Neutral	38	98	75	60
	−*1.5*	−*4.7*	*3.4*	*4.6*
Positive	188	606	258	190
	−*2.7*	−*0.9*	*1.2*	*2.9*
Negative	181	367	103	57
	*6.6*	*1.3*	−*4.0*	−*4.6*
Ambivalent	45	209	88	64
	−*3.6*	*0.8*	*1.1*	*1.7*
Redemption	65	167	54	20
	*1.9*	*1.8*	−*1.0*	−*3.6*
Contamination	17	82	29	14
	−*1.7*	*2.0*	*0.2*	−*1.2*
Sum	534	1529	607	405

	**Text type**			
	**Narrative**	**Chronicle**	**Argument**	**Sum**

Neutral	61	70	140	271
	−*4.4*	−*2.7*	*7.2*	
Positive	367	418	457	1242
	−*5.0*	*0.5*	*4.6*	
Negative	318	181	209	708
	*6.5*	−*4.9*	−*1.7*	
Ambivalent	135	144	127	406
	−*0.7*	*1.1*	−*0.4*	
Redemption	134	135	37	306
	*3.5*	*4.3*	−*7.9*	
Contamination	54	70	18	142
	*0.8*	*4.2*	−*5.1*	
Sum	1069	1018	988	3075

*Rows in italics indicate corrected residuals per cell*.

## Discussion

### Summary

#### Evaluation and narrative text format characterize episodic autobiographical memories

Comparing various characteristics of episodic autobiographical memories, vividness, emotion naming, and narrative text type correlated most with each other. Neither memory type nor detailedness of person, place, or time covaried with vividness as the most uncontroversial indicator of the episodicity of a memory, nor did indicators of detailedness correlate with each other. Detailedness is thus not a correlate of remembering an episode, i.e., a sequence of related actions and events. Static memories of what one’s childhood home looked like or of one’s parents’ characters may be quite detailed without referring to events. Actually detailedness of persons was highest in comments, which apparently contained reflections on a person or relationship. Detailedness of place and time were highest in memory reports covering long life periods and in chronicles, suggesting that not so much memories of very specific events but rather summary reports of long stretches of life, like one’s time in elementary school, require detailed local and temporal background information.

Apparently specific memories (limited to 1 day) are not especially vivid either. Rather it seems to be the sequential way in which events are presented in the text by narrating, and not just summarizing them, which contributes most to the vividness of the memory report. This finding supports the notion that episodic memories at the level of experiencing involve the reliving of an event, which implies most importantly imagining the events in a temporal forward fashion. Temporal sequentiality is one of the two constitutive aspects of narrative. While Tulving’s concept of episodic memories focuses on the subjective experiential level, and narrative focuses on objective textual aspects of memories, both share temporal sequentiality as the core element. However, in memory research this temporally sequential aspect of memories is rarely studied, but mostly inferred once participants state that they remember an event, or it is judged from verbal memory reports, i.e., narratives.

The explicit and communicative nature of a narrative supports following a temporal sequence, whereas tacit remembering requires less temporal order. More probably tacit remembering involves thinking of specific situations, images, or impressions, which in remembering need not be brought into a sequential order. Thus it could well be that it is the narrative text format that more strongly induces a sequential reliving than mere remembering does. This would imply that the sequential aspect of reliving depends less on the memory system, save in some cases like traumatic memories (Rubin et al., 2008), but on the manifest linguistic shaping of the memory.

Aspects of the second constituent of narrative, the evaluation of the events, contributed even more to vividness. There are varying degrees of distancing involved in evaluation. Internal evaluations (Labov and Waletzky, 1967) arise from within the storyline and are made from the points of view of the past protagonists, while external evaluations are by others and from more distanced temporal perspectives. In our study, the naming of emotions substantially correlated with vividness, as did, to a lesser degree, interpretation. For the latter, the more distanced forms of interpreting did not add to vividness over and above the mere naming of emotions. The most distanced form of interpretation, integrating the remembered event into the life story, still correlated positively, if more moderately with vividness.

Finally the mere length of a memory report correlated not only with vividness, but also with emotion, narrativity, interpretation, and life story integration. Thus the length of a memory report seems to be driven by several of these aspects. Taking into account segment length substantially reduced the predictive value of narrative text, without altering its position relative to the other suggested attributes of episodicity. However, length in and of itself is not a variable of primary interest here, but depends on how much an event is narrated and how much it is evaluated. Therefore the prediction of vividness without using segment length reflects the relative influences of text qualities of interest more adequately.

To sum up, as expected narrativity was a stronger predictor of vividness than memory specificity and detailedness. In addition, basic levels of evaluation, i.e., naming emotions, contributed even more to vividness. The comparably small size of the effect of narrative text might be related to the dichotomous coding of narrativity. A rating of the degree of narrativity of memory reports would capture this aspect better and could very well increase its predictive power.

#### Exploration of types of memory reports

The typologies of memory reports revealed some interesting differences, more between text types and less between memory types. First, as expected narratives were more vivid than chronicles and arguments, whereas differences between memory types were minimal. Similarly, narratives were longer than chronicles and arguments, whereas the same trend was weaker for specific memories.

Text types also differed more than memory types with regard to evaluative patterns. As expected, negative events were disproportionately more frequent in specific and narrative memory reports. Redemption sequences were distributed equally across narratives and chronicles as well as specific and generalized events, whereas contaminations sequences were most frequent among chronicles and generalized events. This confirms that negative events tend to motivate more specific and more narrative memory reports than positive or neutral events. In terms of memory and text type, negative memories stuck out. However, memory reports with mixed evaluations, and especially those with a change of fortune stood out in terms of being the most vivid and emotional, the longest, but also the ones that were most often interpreted and integrated into the life story. Again this points to the importance of a narrative structure of memory reports, since narratives normatively contain changes of fortune as events unfold and actions fail or succeed.

### Limitations

The autobiographical memories analyzed in this study were selected as highly self-defining, covered participants’ entire lives, and were narrated by participants from a very wide age range. Therefore they may not be quite comparable to most of the more recent, less self-relevant memories from mostly young adults used in most research on episodic memory. However for example the tendency to produce specific or non-specific memories seems to be comparable when cued with words or asked to narrate self-defining memories (Sumner et al., 2013). In addition, the more personal nature and more diverse age ranges of both memories and participants should render results more relevant for various ages and personally salient autobiographical memories.

The study was correlational. This appears to be adequate if the object of interest is relationships and not causal effects. Memories were not explicitly elicited as memories, but were chunks of continuous life narratives. Thus some parts of life narratives may be produced less with the intention of providing memories but to follow the life line and fill in gaps between memories. Although this probably leads to the production of more non-specific memories than instructions to remember specific episodes, we would argue that regarding autobiographical memory this method provides a broader range of elements of autobiographical remembering more representative of the whole range of ways of autobiographical remembering. In more narrow and controlled studies, specific kinds of episodic memories might be elicited to explore whether defining characteristics are more characteristic under some recall conditions than others. For instance, going beyond the scope of this paper which was limited to autobiographical episodic memories, future studies might compare autobiographical with other kinds of episodic memories such as memories of routine activities, dreams, daydreams, stories known from hearsay, and fictional stories as well as fantasized future scenarios in order to explore whether the autobiographical quality of memories sets them apart from other episodic memories in terms of the defining qualities of interest here.

Only four suggested criteria for the episodicity of memory reports were tested here. Future explorations of the episodicity of memories should also include other aspects of memory reports such as the perspective of mental references, the actual number of narrative clauses, or the completeness of the narrative structure, as well as subjective reports of a sense of remembering vs. knowing, of experiencing that the episode had been personally lived through before, and the intensity of a sense of reliving.

A more serious limitation was the partial semantic overlap between some constructs. This had not been intended when writing the manuals, but sometimes a more operational definition relying on specific indicators proved necessary to achieve the necessary interrater reliability. Specifically there was some overlap between emotion naming and the ratings of vividness and of interpretation. For the mid-range ratings of vividness emotion labels could be one indicator out of several possible. For a rating of 1 in interpretation the presence of emotion labels was sufficient. Although theoretically these definitions do make sense, ideally these two ratings would be defined without operational overlap. Finally participants were well-educated. This may have increased attempts to interpret memories and to integrate them into the life story.

### Implications

#### Not detail, but temporal sequence makes autobiographical memories episodic

The degree of detail in terms of specifications of individuals, place, and time are not essential elements of episodic autobiographical memories. The importance of detail in the memory literature probably stems from memory research’s focus on the veridicality of memory, and the field of episodic memory appears to be slowly shifting away from detailedness (Piolino et al., 2009). For many practical purposes, remembering detail is important, such as when witnessing a crime. Also when remembering important episodes of one’s life veridicality is still a prime issue, since interlocutors do not want to be confronted with an invented life. However it is less the details of an event, but rather the event sequence itself, its causal-motivational internal structure and its meaning that matter. The precise geographical and temporal location is less important than what happened, who did what, why, and with what consequences, and what it tells about the narrator.

Both the temporal sequentiality and a structuring through intentional acts are implied by the choice of the term episode, which denotes a relatively self-contained part of a larger story. Temporal sequentiality is also implied by Tulving’s definition of episodic memories as a reliving of experiences. Also the specificity of memories implies a temporal sequentiality, since it requires events with a temporal extension. But the limitation of the duration of specific memories to 1 day is somewhat arbitrary and reflects a too strong focus on detailedness. The centrality of temporal sequentiality for episodic autobiographical memories suggests that identifying the episodicity of memories objectively in memory reports should rely more on the event character of episodic memories than on atemporal details and dating.

Also, when studying the affective valence of memories, measuring only positive versus negative tone and affective intensity may miss important differences. Memories with conflicting evaluations and or even with a change of fortune, and especially memories of negative events with a happy ending, stand out as especially long, vivid, and emotional as well as involving more interpretation and integration into the life story. Memory reports differ both by the specific emotions involved as well as by the sequence of emotions (Habermas et al., 2009b) and sequence of positive versus negative evaluations (McAdams, 2006).

This insight has consequences for the study of memory in psychopathological states. Thus in depression memories tend not only to be less specific (Williams et al., 2007), but they also differ in their temporal structure. This not only means that in depression memories have less of an internal temporal extension, but also that a change from a positive to a negative evaluation in episodic memories is more frequent (Adler et al., 2006). In addition, depressed patents are more immersed in their past, as shows in a less linear temporal structure of life narratives and less stepping outside past episodes to comment on them (Habermas et al., 2008).

#### The textual quality of episodic memories is narrative

In a way important autobiographical memories require being narrated to oneself as well as to others. If a memory is really relived or, for that case, intensively experienced like a daydream, the events are visually and linguistically narrated to oneself. The normative form of narrative (Labov and Waletzky, 1967) requires not only a temporal ordering of events, but also their causal-motivational structuring so as to understand what happened. Thus a fully episodic autobiographical memory is more than the memory of a specific scene, but requires an interpreted sequence of events and actions.

This is in accordance with an understanding of the ability to remember as being culturally socialized (Nelson and Fivush, 2004; Habermas et al., 2010; Fivush et al., 2011) and of memories as being culturally framed, put into and formed by text, and communicated in language (Markowitsch and Staniloiu, 2011). Thus more attention should be paid to the actual textual and narrative qualities of autobiographical memories. Here we used a rating and global coding methodology which is more typical for memory research than for linguistic analyses. We have demonstrated narrative analyses of the textual surface of autobiographical memories of a more differentiated linguistic kind elsewhere (e.g., Habermas et al., 2009b), which might well increase the correlation of narrativity measures with vividness.

#### Autobiographical episodic memories are not only relived and narrated, they are also evaluated

There are varying degrees of the immediacy of evaluating, ranging from more affective to more cognitive modes, from perceptions to emotions and thoughts in the past to those from a present or hypothetical perspective, from immediate reactions to more effortful and complex interpretative efforts. This dimension implies varying degrees of narrative distancing from the past event. Past perceptions and past direct speech are very close to past experiences (Habermas et al., 2008). Also the naming of emotions is relatively close to past events and therefore correlates highly with vividness.

Complex form of evaluation such as interpretation and autobiographical reasoning are more distanced forms of evaluation. Autobiographical reasoning establishes logical links between a past event and other, distant parts of life or with the narrator’s personality and its development (Habermas and Bluck, 2000; Habermas, 2011). What we termed life story integration here, the mere mentioning of other parts of life or of personality, is a simple measure of autobiographical reasoning. Autobiographical reasoning goes beyond mere remembering by establishing the meaning of the past event for who the remembering person was and is. The ability for creating coherent life narratives and for autobiographical reasoning, i.e., a specifically biographical view on events, develops between late childhood and early adulthood, as was demonstrated in two studies (Bohn and Berntsen, 2008; Habermas and de Silveira, 2008). First studies of the location of brain activities correlated with autobiographical reasoning have also been undertaken (D’Argembeau et al., 2008, in press). In this study, interpretation and autobiographical reasoning were related to changes of fortune in the memory and to its vividness and emotionality.

Episodic memories of biographically non-salient events do not evoke much of an interpretation or autobiographical reasoning, which therefore can be ignored without a great loss. However the more potentially personally significant a past event is, the more autobiographical reasoning becomes part of the process of remembering, and the more it should be included in studies of episodic autobiographical memories.

## Conflict of Interest Statement

The authors declare that the research was conducted in the absence of any commercial or financial relationships that could be construed as a potential conflict of interest.

## Appendix

### Male participant, 8 years, total of six segments, segment 6 “flea market”

“Now I have to think what else. There was a flea market now. That was very exciting. That was fun. I sold some stuff.”

Ratings: vividness 2, detailedness – person 0, place 1, time 2, specific memory, chronicle, emotion 3, interpretation 1, life story integration 0, valence 1, 0 months old memory, 5 propositions.

### Male participant, 20 years, total of 22 segments, segment 21 “Father doesn’t know everything”

“But already in 7th grade I started asking questions, which my father couldn’t give an answer to, and then it was “I don’t know it”. But then I was thinking by myself, “Why, why doesn’t he know it?” I mean, that’s when I first noticed, that my father doesn’t know everything, that he is also only human, that he is a real normal person. Before then it was always like, my Dad is my big role model.”

Ratings: vividness 3, detailedness – person 2, place 0, time 1, extended memory, narrative, emotion 0, interpretation 2, life story integration 1, valence 2, 103 months old memory, 13 propositions.

### Female participant, 60 years, 37 segments, segment 24 “Father remarries”

“That year my father married for a second time. And that was quite incisive because he totally focussed on his new wife and family, such that I – had done my part, and we have no contact till today. I can’t accept letting myself be hurt anymore. It was like, he was like totally changed, as if what I did, didn’t count for anything anymore, I had passed my youth, my early adulthood, when other women start a family of their own, I spent that time with my father. Therefore for me it was like he gave me a kick. We had been very close, almost like a couple without sex. We had even been addressed as husband and wife. And then he just remarried, very fast after my mother had died, which I could not understand. But well, that’s the way it went.”

Ratings:, vividness 2, detailedness – person 1, place 0, time 1, specific memory, argument, emotion 2, interpretation 2, life story integration 2, valence −1, 326 months old memory, 21 propositions.

## References

[B1] Adler J. M. Kissel E. C. McAdams D. P. (2006). Emerging from the CAVE: attributional style and the narrative study of identity in midlife adults. Cognit. Ther. Res. 30, 39–51 10.1007/s10608-006-9005-1

[B2] Barsalou L. W. (1988). “The content and organization of autobiographical memories,” in Remembering Reconsidered: Ecological and Traditional Approaches to the Study of Memory, eds Neisser U. Winograd E. (New York: Cambridge University Press), 193–243

[B3] Bluck S. Habermas T. (2000). The life story schema. Motiv. Emot. 24, 121–147 10.1023/A:1005615331901

[B4] Bohn A. Berntsen D. (2008). Life story development in childhood. Dev. Psychol. 44, 1135–1142 10.1037/0012-1649.44.4.1135 18605840

[B5] Bruner J. (1990). Acts of Meaning. Cambridge, MA: Harvard University Press

[B6] Conway M. A. (1990). Autobiographical Memory. Buckingham: Open University Press

[B7] Conway M. A. (2009). Episodic memories. Neuropsychologia 47, 2305–2313 10.1016/j.neuropsychologia.2009.02.003 19524094

[B8] Conway M. A. Pleydell-Pearce C. W. (2000). The construction of autobiographical memories in the self-memory system. Psychol. Rev. 107, 261–288 10.1037/0033-295X.107.2.261 10789197

[B9] Conway M. A. Singer J. A. Tagini A. (2004). The self and autobiographical memory: correspondence and coherence. Soc. Cogn. 22, 491–529 10.1521/soco.22.5.491.50768

[B10] D’Argembeau A. Cassol H. Philipps C. Balteau E. Salmon E. van der Linden M. (in press). Brains creating stories of selves: the neural basis of autobiographical reasoning. Soc. Cogn. Affect. Neurosci. 10.1093/scan/nst028 23482628 PMC4014101

[B11] D’Argembeau A. Fevers D. Maierus S. Collette F. van der Linden M. Maquet P. (2008). Self-reflection across time: cortical midline structures differentiate between present and past. Soc. Cogn. Affect. Neurosci. 3, 244–252 10.1093/scan/nsn020 19015116 PMC2566769

[B12] Diel V. Elian C. Weber A. (2007). Manual zur Thematischen Segmentierung Autobiographischer Erzählungen [Manual for Segmenting Autobiographical Narratives]. Unpublished manual, Goethe University, Frankfurt

[B13] Diel V. Heberer J. Kraaz A. Mahmoudi A. Streck L. Habermas T. (2009). Manual zum Kodieren von Lebendigkeit, Emotionsbenennungen, Interpretation, Integration in Die Lebensgeschichte, Detailliertheit, Valenz, Gedächtnistypen und Textsorten in Segmenten Autobiographischer Erzählungen [Manual for Coding Vividness, Emotion Naming, Interpretation, Life Story Integration, Detailedness, Valence, Memory Type, and Text Type in Segmented Autobiographical Narratives]. Unpublished manual, Goethe University, Frankfurt

[B14] Fitzgerald J. M. (1988). Vivid memories and the reminiscence phenomenon: the role of a self narrative. Hum. Dev. 31, 261–273 10.1159/000275814

[B15] Fivush R. Habermas T. Waters T. Zaman W. (2011). The development of autobiographical memory. Int. J. Psychol. 46, 321–345 10.1080/00207594.2011.596541 22044305

[B16] Habermas T. (2011). “Autobiographical reasoning: mechanisms and functions,” in The Development of Autobiographical Reasoning in Adolescence and Beyond. New Directions in Child and Adolescent Development, ed. Habermas T. (San Francisco: Jossey-Bass), 131, 1–17 10.1002/cd.285 21387528

[B17] Habermas T. Berger N. (2011). Retelling everyday emotional events: condensation, distancing, and closure. Cogn. Emot. 25, 206–219 10.1080/02699931003783568 21432668

[B18] Habermas T. Bluck S. (2000). Getting a life: the development of the life story in adolescence. Psychol. Bull. 126, 748–769 10.1037/0033-2909.126.5.748 10989622

[B19] Habermas T. de Silveira C. (2008). The development of global coherence in life narratives across adolescence: temporal, causal, and thematic aspects. Dev. Psychol. 44, 707–721 10.1037/0012-1649.44.3.707 18473638

[B20] Habermas T. Diel V. (2010). The emotional impact of loss narratives: event severity and narrative perspectives. Emotion 10, 312–323 10.1037/a0018001 20515221

[B21] Habermas T. Diel V. Welzer H. (in press). Lifespan trends of autobiographical remembering: episodicity and search for meaning. Conscious. Cogn. 10.1016/j.concog.2013.07.010 23948342

[B22] Habermas T. Ehlert-Lerche S. de Silveira C. (2009a). The development of the temporal macrostructure of life narratives across adolescence: beginnings, linear narrative form, and endings. J. Pers. 77, 527–560 10.1111/j.1467-6494.2008.00557.x 19220721

[B23] Habermas T. Meier M. Mukhtar B. (2009b). Are specific emotions narrated differently? Emotion 9, 751–762 10.1037/a0018002 20001120

[B24] Habermas T. Köber C. (in press). “Autobiographical reasoning is constitutive for narrative identity: the role of the life story for personal continuity,” in The Oxford Handbook of Identity Development, eds McLean K. C. Syed M. (Oxford: Oxford University Press).

[B25] Habermas T. Negele A. Brenneisen Mayer F. (2010). Honey, you’re jumping about – mothers’ scaffolding of their children’s and adolescents’ life narration. Cogn. Dev. 25, 339–351 10.1016/j.cogdev.2010.08.004

[B26] Habermas T. Ott L. M. Schubert M. Schneider B. Pate A. (2008). Stuck in the past: negative bias, explanatory style, temporal order, and evaluative perspectives in life narratives of clinically depressed individuals. Depress. Anxiety 25, E121–E132 10.1002/da.20389 17957809

[B27] Hemphill J. F. (2003). Interpreting the magnitudes of correlation coefficients. Am. Psychol. 58, 78–80 10.1037/0003-066X.58.1.78 12674822

[B28] Kopelman M. D. Wilson B. A. Baddeley A. D. (1989). The autobiographical memory interview: a new assessment of autobiographical and personal semantic memory in amnesic patients. J. Clin. Exp. Neuropsychol. 11, 724–744 10.1080/01688638908400928 2808661

[B29] Labov W. Waletzky J. (1967). “Narrative analysis: oral versions of personal experience,” in Essays on the Verbal and Visual Arts. Proceedings of the 1966 Annual Spring Meeting of the American Ethnological Society, ed. Helm I. (Seattle, WA: University of Washington Press), 12–44

[B30] Levine B. Svoboda E. Hay J. F. Winocur W. Moscovitch M. (2002). Aging and autobiographical memory: dissociating episodic from semantic memory. Psychol. Aging 17, 677–689 10.1037/0882-7974.17.4.677 12507363

[B31] Lilgendahl J. P. McAdams D. P. (2011). Constructing stories of self-growth: how individual differences in patters of autobiographical reasoning relate to well-being. J. Pers. 779, 391–428 10.1111/j.1467-6494.2010.00688.x 21395593 PMC3386782

[B32] Linde C. (1993). Life Stories. Oxford: Oxford University Press

[B33] Markowitsch H. J. Staniloiu A. (2011). Memory, consciousness, and the self. Conscious. Cogn. 20, 16–39 10.1016/j.concog.2010.09.005 20951059

[B34] McAdams D. P. (2006). The Redemptive Self. Oxford: Oxford University Press

[B35] Neisser U. (1982). Memory Observed: Remembering in Natural Contexts. New York: Freeman

[B36] Neisser U. (1986). “The nested structure of autobiographical memory,” in Autobiographical Memory, ed. Rubin D. C. (Cambridge: Cambridge University Press), 71–81

[B37] Nelson K. Fivush R. (2004). The emergence of autobiographical memory: a social cultural developmental theory. Psychol. Rev. 111, 486–511 10.1037/0033-295X.111.2.486 15065919

[B38] Nelson K. L. Moskovitz D. J. Steiner H. (2008). Narration and vividness as measures of event-specificity in autobiographical memory. Discourse Process. 45, 195–209 10.1080/01638530701792891

[B39] Piolino P. Desgranges B. Eustache F. (2009). Episodic autobiographical memories over the course of time: cognitive, neuropsychological and neuroimaging findings. Neuropsychologia 47, 2314–2329 10.1016/j.neuropsychologia.2009.01.020 19524095

[B40] Robinson J. A. (1986). “Autobiographical memory: a historical prologue,” in Autobiographical Memory, ed. Rubin D. C. (Cambridge: Cambridge University Press), 9–24

[B41] Rosenbaum S. Gilboa A. Levine B. Winocur G. Moscovitch M. (2009). Amnesia as an impairment of detail generation and binding: evidence from personal, fictional, and semantic narratives in K. C. Neuropsychologia 47, 2181–2187 10.1016/j.neuropsychologia.2008.11.028 19100757

[B42] Rosenthal G. (1995). Erlebte und Erzählte Lebensgeschichte [The Lived and Narrated Life Story]. Frankfurt: Campus

[B43] Rubin D. C. Berntsen D. Bohni M. K. (2008). A memory-based model of posttraumatic stress disorder: evaluating basic assumptions underlying the PTSD diagnosis. Psychol. Rev. 115, 985–1011 10.1037/a0013397 18954211 PMC2762652

[B44] Rubin D. C. Wetzler S. E. Nebes R. D. (1986). “Autobiographical memory across the life span,” in Autobiographical Memory, ed. Rubin D. C. (Cambridge: Cambridge University Press), 202–221

[B45] Sumner J. A. Mineka S. McAdams D. P. (2013). Specificity in autobiographical narratives correlates with performance on the Autobiographical Memory Test and prospectively predicts depressive symptoms. Memory 21, 646–656 10.1080/09658211.2012.746372 23240988 PMC3609943

[B46] Tulving E. (1972). “Episodic and semantic memory,” in Organization of Memory, eds Tulving E. Donaldson W. (New York: Academic Press), 381–402

[B47] Tulving E. (1983). Elements of Episodic Memory. New York: Oxford University Press

[B48] Tulving E. (1985). Memory and consciousness. Can. Psychol. 26, 1–12 10.1037/h0080017

[B49] Tulving E. (2002). Episodic memory: from mind to brain. Annu. Rev. Psychol. 53, 1–25 10.1146/annurev.psych.53.100901.135114 11752477

[B50] Waters T. Bauer P. Fivush R. (2013). Autobiographical Memory Functions, Served by Multiple Event Types. Unpublished manuscript, Department of Psychology, Emory University, Atlanta, GA

[B51] Wheeler M. A. Stuss D. T. Tulving E. (1997). Toward a theory of episodic memory: the frontal lobes and autonoetic consciousness. Psychol. Bull. 121, 331–354 10.1037/0033-2909.121.3.331 9136640

[B52] Williams J. M. G. Barnhofer T. Crane C. Herman D. Raes F. Watkins E. (2007). Autobiographical memory specificity and emotional disorder. Psychol. Bull. 133, 122–148 10.1037/0033-2909.133.1.122 17201573 PMC2834574

[B53] Willoughby K. A. Desrocher M. Levine B. Rovet J. F. (2012). Episodic and semantic autobiographical memory and everyday memory during late childhood and early adolescence. Front. Psychol. 3:53 10.3389/fpsyg.2012.00053 22403560 PMC3289112

